# Management and survival of patients with cancer of unknown primary discussed by a French national multidisciplinary tumour board: a retrospective analysis

**DOI:** 10.1016/j.lanepe.2025.101524

**Published:** 2025-11-07

**Authors:** Célia Dupain, Nicolas Jacquin, Aurélien Latouche, Zoé Nevière, Pierre Gestraud, Abderaouf Hamza, Kenza Nedara, Vincent Cockenpot, Janick Selves, Yves Allory, Laëtitia Chanas, Maud Milder, Isabelle Soubeyran, Hélène Blons, Anna Patrikidou, Axel de Bernardi, Julien Masliah-Planchon, Odette Mariani, Etienne Rouleau, Fabienne Escande, Sandrine Boyault, Pierre Saintigny, Florence de Fraipont, Pierre Blanc, Jennifer Wong, Camille Tlemsani, Isabelle Guillou, Julie Flavius, Noemie Fuentealba, Maud Kamal, Ivan Bièche, Nicolas Servant, Christophe Le Tourneau, Sarah Watson

**Affiliations:** a Department of Drug Development and Innovation (D3i), Institut Curie, Paris, France; b Department of Medical Oncology, Institut Curie, Paris, France; c Department of Medical Oncology, Institut Godinot, Reims, France; d INSERM U1331, Institut Curie, Saint-Cloud, France and Paris-Saclay University, Paris, France; e Conservatoire National des Arts et Metiers, Paris, France; f Department of Medical Oncology, Centre François Baclesse, Caen, France; g INSERM U1331, Curie CoreTech Bioinformatics (CUBIC), Institut Curie Research Center, Mines ParisTech, Paris, France; h Department of Genetics, Institut Curie, Paris, France; i INSERM U1016, CNRS UMR8104, Université Paris Cité, CARPEM, Institut Cochin, Paris, France; j Department of Pathology, Institut Curie, Paris, France; k Department of Pathology, Institut Universitaire du Cancer Oncopole University Hospital of Toulouse, 31059, Toulouse, France; l Université Toulouse III-Paul Sabatier, F-31000, Toulouse, France; m Data Office, Institut Curie, Paris, France; n Department of Pathology, Institut Bergonié, Bordeaux, France; o Institut du Cancer Paris Carpem, APHP Centre, Department of Biology, Pharmacogenomics and Molecular Oncology Unit, Hôpital Européen Georges Pompidou, Paris, France; p Department of Cancer Medicine, Gustave Roussy Cancer Center, Université Paris-Saclay, Villejuif, France; q Department of Medical Oncology, Centre Léon Bérard, Lyon, France; r Center of Biological Ressources, Institut Curie, Paris, France; s Biology and Pathology Department, Cancer Genetic Service, Gustave Roussy Cancer Center, U981, Université Paris-Saclay, Villejuif, France; t CHRU Lille, Lille, France; u Department of Translationnal Research and Innovation, Centre de Recherche en Cancérologie de Lyon CRCL, INSERM U1052, CNRS UMR 5286, Centre Léon Bérard, UCBL1, Lyon, France; v CHU Grenoble, France; w Laboratoire SeqOIA, France; x Department of Medical Oncology, Hôpital Cochin, APHP Centre, Université Paris Cité, Paris, France; y INSERM U1339 CNRS UMR3666, PSL Research University, Institut Curie Research Center, Paris, France

**Keywords:** Cancer of unknown primary, Multidisciplinary tumour board, Next generation sequencing, Personalized medicine, Survival

## Abstract

**Background:**

Recent clinical trials have shown that molecularly-guided treatments can improve survival in patients with cancers of unknown primary (CUP). However, the feasibility and clinical benefit of these treatments for CUP in a real-life setting remain uncertain. In France, a national multidisciplinary tumour board dedicated to patients with CUP (CUP MTB) was created in 2020, with the aims of coordinating pathological and molecular diagnostic analyses and providing a centralised expertise for therapeutic orientation. This study aimed at evaluating the diagnostic and therapeutic impact of the CUP MTB on patients with CUP in a national real-life setting.

**Methods:**

Patient and tumour characteristics, treatments and outcomes are collected prospectively. This study reports the diagnostic and therapeutic impact of all patients discussed in CUP_MTB between July 2020 and December 2023. The diagnostic impact was defined as the identification of a putative tissue of origin, and the initiation of a MTB–oriented treatment. Overall survival was estimated using the Kaplan–Meier method, and hazard ratios were calculated using Cox proportional hazard models.

**Findings:**

A total of 246 CUP patients were referred to CUP_MTB (124 females and 122 men); 187 (76%) underwent pathological and molecular characterizations as recommended by the MTB. Tumour profiling enabled the identification of a putative tissue of origin (TOO) in 130/187 (70%) patients. The most frequent TOO were gastrointestinal (n = 29; 22%), lung (n = 22; 17%), breast (n = 21; 16%), and kidney (n = 19; 15%). 149 (61%) patients received a treatment based on MTB recommendation. 111/149 (74.5%) patients received MTB-oriented treatment, including systemic treatment oriented towards the putative TOO (n = 95, 63.8%), or treatment directed towards a targetable molecular alteration (n = 16, 10.7%). 38 (25.5%) patients for whom no MTB-oriented treatment could be recommended were treated with empiric treatment according to international guidelines. The median overall survival of patients treated with MTB-oriented treatment was 18.6 (IQR = 12.0) months, compared to 11.0 (IQR = 10.5) months in patients with empiric treatment (HR = 0.61, 95% CI 0.38–0.98, p = 0.04).

**Interpretation:**

Integration of clinical, pathological and molecular data within an expert MTB is feasible in a real-life setting, enables access to molecularly guided treatments and improves survival for a large proportion of CUP patients. Our findings highlight the benefits of dedicated MTB and reference centres to improve the management of CUP.

**Funding:**

10.13039/501100010463Institut Curie and the 2025 French Genomic Medicine Initiative.

**Research in context**

**Evidence before this study**

We searched PubMed, Embase, and Web of Science from database inception up to March 2024 without language restrictions using combinations of the terms “cancer of unknown primary”, “CUP”, “tissue of origin”, “molecular profiling”, “agnostic treatment”, “MTB”, and “precision oncology”. References from key reviews and clinical trials were also screened. Studies were included if they reported on the diagnosis, molecular characterization, or treatment of CUP, regardless of patient performance status or setting. The evidence highlighted the poor prognosis of CUP, with standard treatment remaining largely limited to empirical chemotherapy. Two main strategies have emerged in the last decade^1^: tissue-gnostic approaches aiming to identify and treat the most probable tissue of origin using molecular classifiers, and^2^ tissue-agnostic strategies based on actionable genomic alterations. However, the quality of this evidence was often limited by a high risk of selection bias, with prior studies relying on highly selected patient populations within clinical trials. Furthermore, the heterogeneity of study designs and patient cohorts precluded a formal meta-analysis. Few studies evaluated these approaches in real-world, unselected cohorts, and none incorporated centralized, expert-driven multidisciplinary evaluation of patients with CUP at the national level.

**Added value of this study**

This is the first nationwide study evaluating a multidisciplinary tumour board dedicated to CUP in a real-world setting. By analysing 246 consecutive patients referred to the French national CUP_MTB between 2020 and 2023, our study demonstrates the feasibility and clinical value of an integrative diagnostic strategy that combines centralized pathological review, transcriptomic and genomic profiling, and multidisciplinary expert consensus. A likely tissue of origin was identified in 70% of fully characterized patients, and actionable alterations were found in 59%. Crucially, our findings show that treatment recommendations based on this integrative MTB model led to a clinically meaningful improvement in overall survival compared to standard empirical therapy. Our study confirms that precision medicine for CUP is achievable and impactful outside of clinical trial settings, even in a heterogeneous and high-risk population.

**Implications of all the available evidence**

Combined with existing evidence, our findings provide a strong rationale for the widespread implementation of national expert MTBs and integrated diagnostic workflows for patients with CUP. Centralizing pathological and molecular evaluation ensures equitable access to precision oncology tools and improves both diagnostic yield and survival outcomes. This integrative model provides compelling evidence to redefine the conventional CUP classification, suggesting that future guidelines should evolve to account for molecular and expert-based stratification. Therefore, policymakers and healthcare systems should prioritize access to expert multidisciplinary discussions early in the diagnostic pathway for CUP. Further research is needed to optimize turnaround times, broaden access to targeted therapies, and prospectively evaluate cost-effectiveness of such national strategies.

## Introduction

Cancers of Unknown Primary (CUP) refers to a heterogeneous group of malignancies characterized by the presence of metastases without an established primary site despite proper diagnostic work-up.^1^^,^^2^ Although CUP account for only 2%–5% of all malignancies, they represent the fourth leading cause of cancer-related mortality due to their dismal prognosis.^3^ Up to 85% of patients with CUP belong to a subset with unfavourable prognosis, characterized by a median overall survival below one year.^4^ Standard of care for this group consists in empiric non-specific platinum-based chemotherapy and has not evolved over the last decades.^5^ However, the recent development of precision medicine informed by molecular profiling has increased the ability to determine the culprit primary or to identify targetable molecular alterations in patients with CUP.^6^, ^7^, ^8^ Consequently, two new approaches have emerged. First, the tissue-gnostic approach proposes to treat patients with a tailored therapy based on the most probable primary site. This strategy implies the identification of a tissue of origin using molecular prediction tools based on gene expression profiling, miRNA expression, DNA alterations, or DNA methylation in addition to pathological analyses and other classical means.^9^, ^10^, ^11^, ^12^, ^13^, ^14^ In line with this approach, several machine learning-based classifiers have been developed and validated to determine the most likely primary site based on pathological or molecular data of CUP metastases.^15^, ^16^, ^17^, ^18^, ^19^, ^20^ Second, the tissue-agnostic approach aims to treat patients with targeted therapies or immunotherapies selected according to the molecular alterations present in the tumour, even if a culprit primary could not be established.^21^ If the first studies evaluating tissue-gnostic strategies failed to show their superiority compared to the classical management of CUP based on empiric chemotherapy, recent studies have shown improved outcomes using molecularly guided therapy.^22^, ^23^, ^24^, ^25^, ^26^ These controversial results can be explained at least in part by 1) the selective design of trials that evaluated either the agnostic or the gnostic approaches in isolation; 2) the determination of the primary site based solely on molecular findings; and 3) the heterogeneity and intrinsic dismal prognosis of the tumour subtypes included. Moreover, these trials only included patients with good performance status and selected histologies, and the feasibility and benefit of precision medicine for patients with CUP in a real-life setting remains poorly documented.^27^ In addition, patient access to pathological and molecular expertise for CUP is highly heterogeneous across centres, and most patients managed in tertiary hospitals do not benefit from optimal diagnostic work-up.

We hypothesize that the management of patients with CUP could be improved by an integrative approach that incorporates clinical, pathological and molecular information to better identify a likely tissue of origin and propose an adapted agnostic or gnostic therapeutic strategy. Thus, a national Multidisciplinary Tumour Board for CUP (CUP_MTB) was created in July 2020 to implement and evaluate this integrative strategy and to facilitate access to pathological expertise and molecular analyses for all patients with CUP in France.^28^ CUP_MTB consists in a bimonthly meeting where diagnostic and therapeutic management of patients with CUP across the country can be discussed by expert pathologists, oncologists and molecular biologists, at any stage of the disease, and where complementary molecular analyses are coordinated, using local and national sequencing platforms.^29^ In this study, we report the diagnostic and therapeutic management of a real-life population of consecutive patients with CUP discussed during CUP_MTB between July 2020 and December 2023. We show that a centralized and integrative characterization by a reference team, though challenging, is feasible and improves the management and clinical outcomes of patients with CUP in a real-life setting.

## Methods

Detailed experimental procedures are described as Supplementary Material and Methods.

### Patient selection and MTB organization

CUP_MTB was established in 2020 and aims at centralizing clinical data, pathological and molecular analyses to guide diagnosis and therapy for patients with CUP at the French national level.

From 2020 to 2023, the MTB met every two weeks via videoconference and gathered at least two medical oncologists, two pathologists, two geneticists and two medical biologists, all experts in CUP and affiliated to high-volume academic centres. Patients referred to the MTB are discussed on medical oncologists’ requests, at diagnosis or after previous treatments, and from various centres across France. Our MTB is part of the French Genomic Medicine Initiative 2025 (FGMI2025).^29^ The overall MTB workflow and organization are further detailed in Supplementary Figure S1 and Supplementary Methods. CUP_MTB is organized in two consecutive steps: in CUP_MTB_1, experts validate the diagnosis of CUP according to the current ESMO guidelines,^5^ and recommend additional clinical, pathological and/or molecular analyses depending on available tumour material. The results of all recommended analyses are later discussed in CUP_MTB_2, during which diagnosis and therapeutic recommendations are being made. CUP_MTB provides therapeutic orientation, but treatment decision is made by referent clinicians at any time.

All patients discussed at least once during CUP_MTB between 2020 and 2023 were included in this study. Clinical, molecular, pathological and survival data were collected from medical records and included in a nation-wide secured database. Informed consent or patient non-objection for clinical data collection and analysis, tumour samples and molecular analysis were obtained from all patients enrolled in this study. The study was approved by the Institut Curie review board “Comité de Révision Interne” under the number DATA220203.

### Variables collection

Clinical and demographic data collected included: i) pre-MTB data, including age, gender, addressing centre, metastatic sites, previous imaging results, family history, ECOG performance status, prior treatments, and pathology and molecular analyses reports, ii) MTB data, including samples selected, molecular analyses performed, genomic alterations identified, and MTB conclusions and recommendations, and iii) MTB follow-up data, including treatment received after the MTB, response to treatment according to RECIST1.1 reviewed by a medical oncologist, and survival outcome with date of progression defined as first progression associated with treatment received after CUP_MTB_2 according to RECIST1.1, date of last follow-up, and date of death.

All data were collected after each MTB into a clinico-biological database on a dedicated secured eCRF (REDCap^30^^,^^31^) by the MTB coordination team. Pre-MTB data were gathered by the referring oncologist, who completed a dedicated patient registration form with the patient's medical history and shared it with the MTB members to be included in the database.

MTB-related data were collected from patients' medical files. From August 2024 to November 2024, data were retrospectively verified, updated and completed from patients’ medical files by the MTB coordination team, supervised by two medical oncologists (SW and NJ). All data were obtained from medical records, including pathological and molecular data. A description of all the pathological and molecular analyses performed within CUP_MTB are detailed in Supplementary Material and Methods.

### Statistical analyses

Extreme values, means and standard deviations were reported for continuous variables. Categorical variables were described as frequencies (percentages). Overall survival from CUP_MTB_1 (OS_1) was defined as the time from CUP_MTB_1 to death from any cause. Overall survival from CUP_MTB_2 (OS_2) was defined as the time from CUP_MTB_2 to death of any cause. For non-deceased patients, data were censored at the time of the last visit. Kaplan-Meir method was used to estimate the survival curves and survivals curves were compared using the log-rank test. Prognostic factors were identified using Cox proportional hazard model (Univariable or Multivariable). Hazard-ratios were quoted with their 95% confidence intervals [95% CI]. All tests were two-sided, and the threshold for statistical significance was set to p < 0.05. All statistical analyses were performed using R (version 4.4.1).

### Role of funding source

The research was supported by the Institut Curie, which provided personnel, infrastructure, and equipment, and by the 2025 French Genomic Medicine Initiative, which funded personnel and molecular analyses. The funders had no role in study design, data collection, data analysis, data interpretation, decision to publish, or preparation of the manuscript.

## Results

### Study populations

Between June 2020 and December 2023, 277 consecutive patients with a presumed diagnosis of CUP and referred from all regions of France were discussed in CUP_MTB_1 (Supplementary Figure S2A). The number of cases referred to the MTB increased during the study period, from a mean of 4 new cases/month in 2021 to a mean of 12 new cases/month in 2023. Most patients were referred to the MTB by their medical oncologist (260/277, 94%). Patients were mostly treated in comprehensive cancer centres (n = 136/277; 49%), followed by university hospitals (n = 88/277; 32%), regional hospitals (n = 35/277; 13%), for-profit private institutions (n = 14/277; 5%) and non-profit private institutions (n = 4/277; 1%) (Supplementary Figure 2B).

**Fig. 1:**
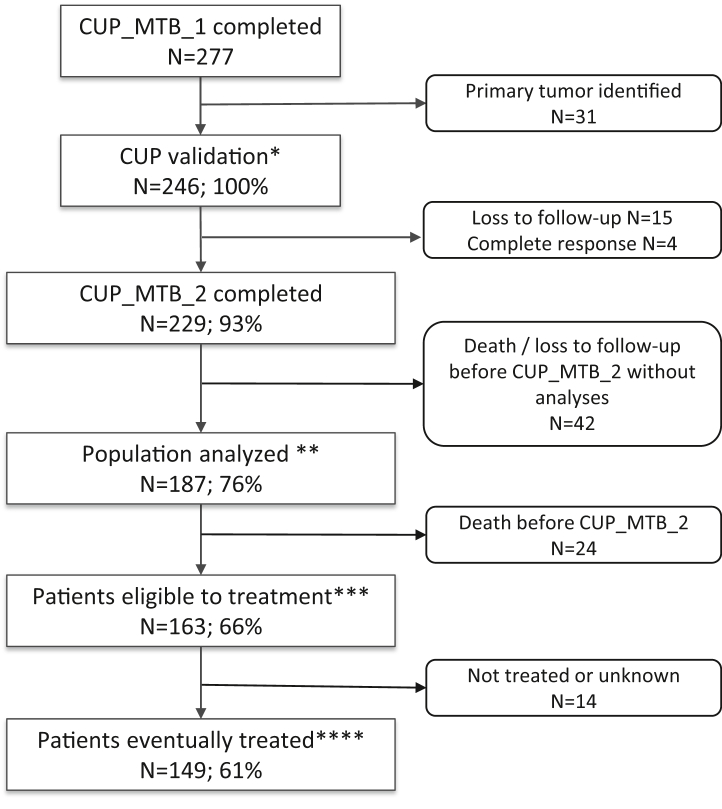
**Study flowchart**. ∗ CUP_total_population; ∗∗ CUP_diagnostic_population; ∗∗∗ CUP_therapeutic_population; ∗∗∗∗ CUP_treated_population.

CUP diagnosis, defined as metastatic cancer with absence of detectable primary tumour after the standard diagnostic work-up described in the current ESMO guidelines,^5^ was confirmed by the MTB for 246 patients (CUP_total_population) (Supplementary Table S1 and Fig. 1). At time of data analysis, CUP_MTB_2 was completed for 229/246 (93%) patients. Among them, 42 patients died before any additional analyses could be performed. Thus, complete pathological and/or molecular tumour profiles were available for discussion in CUP_MTB_2 and diagnostic orientation could be proposed for 187/246 (76%) patients (CUP_diagnostic_population). A total of 163/246 (66%) patients were alive at time of CUP_MTB_2 and eligible for treatment (CUP_therapeutic population). Among them, 149/246 (60%) received a treatment following CUP_MTB_2 (CUP_treated_population).

### CUP patient characteristics

The main characteristics of CUP_total_population patients are described in Table 1. Median age at diagnosis was 61 (IQR = 19), and 50% were female. Performance status (ECOG) at diagnosis was >1 in 28% of patients. 113/246 (46%) were past or active smokers and 60/246 (24%) had a personal history of cancer. The median number of metastatic sites at diagnosis was 2 (range 1–7). The most frequent metastatic sites were lymph nodes (n = 150/246, 61%), bone (n = 89/246, 36%), lung (n = 70/246, 28%), and liver (n = 61/246, 25%).

**Table 1:** Patient characteristics (CUP_total_population).

Patient characteristics	n = 246	%
Sex		
Female	124	50
Male	122	50
Age at diagnosis (years)		
Median	61	
Range	18–90	
Risk factors		
Past or active Smokers		
Yes	113	46
No	120	49
Unknown	13	5
Alcohol use		
Yes	43	17
No	188	76
Unknown	15	6
Personal history of cancer		
Yes	60	24
No	185	75
Unknown	1	0
ECOG Performance Status at diagnosis		
0	96	39
1	82	34
2	48	20
3	6	2
4	1	–
Unknown	13	5
Number of different metastatic sites per patient		
Median	2	
Range	1–7	
Metastatic sites at diagnosis		
Lymph nodes	150	61
Bone	89	36
Lung	70	28
Liver	61	25
Peritoneal carcinomatosis	49	20
Brain	19	8
Histological types		
Undifferenciated Carcinoma	103	42
Adenocarcinoma	96	40
Squamous Cell Carcinoma	26	11
Other	21	8
Molecular analysis before CUP_MTB		
DNA sequencing	131	53
RNA sequencing	60	24
Prognostic group at diagnosis according to ESMO guidelines		
Favourable	27	11
Unfavourable	219	89
TOO suspected before CUP_MTB		
No	116	47
Yes	130	53
Lung	27	11
Gastrointestinal	42	17
Breast	14	6
Kidney	12	5
Gynecological tract	11	4
Sarcoma	7	3
Urinary tract	4	2
Head and Neck	4	2
Skin	4	2
Prostate	2	1
Other	3	1
Number of previous treatment lines		
0	73	30
1	108	44
2–3	56	23
>3	9	4

Local pathological characterization of at least one tumour lesion was available at CUP_MTB_1 for all patients. The most frequent histological subtypes were undifferentiated carcinoma (n = 103/246, 42%), adenocarcinoma (n = 96/246, 40%), and squamous cell carcinoma (n = 26/246, 11%). Molecular profiling performed locally before CUP_MTB_1 by targeted DNA sequencing and RNA sequencing was available for 131/246 (53%) and 60/246 (24%) patients, respectively. A specific tissue of origin (TOO) was suspected by referent physicians before CUP_MTB_1 in 130/246 (53%) patients. The most frequent suspected TOO were lung (n = 27/246, 11%), gastrointestinal (n = 42/246, 17%), breast (n = 14/246, 6%) and kidney (n = 12/246, 5%) cancers. Altogether, 219/246 (89%) patients presented with CUP of the unfavourable group according to the definition provided in the current ESMO guidelines.^5^

The median time from diagnosis to CUP_MTB_1 was 2 months (range 0–91, IQR = 6 months, IDR 10–90% = 16.5 months). Thirty percent (73/246) of patients were discussed at CUP_MTB_1 before initiating any treatment, and 173/246 had started at least one line of systemic treatment before CUP_MTB_1. The median number of previous treatment lines was 1 (range 0–5, IQR = 2), with a majority of patients having previously received only one treatment line (118/246, 48%) (Supplementary Table S1 and Table 1). In these later cases, systemic treatment was oriented toward a putative tissue of origin in 63/118 (53%) cases, whereas 45/118 (38%) patients had received empiric platinum-based chemotherapy.

### Complementary analyses recommended by CUP_MTB

CUP_MTB_1 recommended complementary analyses in 245 out of the 246 patients within the CUP_total_population (Supplementary Table S2), including imaging studies in 35 (14%) cases, pathological assessment with additional immunohistochemical stainings or centralized pathological review in 150 (61%) cases, and molecular analyses in 244 (99%) cases. Complementary examinations requested by CUP_MTB_1 were available for discussion during CUP_MTB_2 for 17/2 (40%) radiological exams recommended, 96/151 (64%) pathological exams and for 301/633 (48%) molecular exams recommended.

The median time from CUP_MTB_1 to CUP_MTB_2 was 3 months (range 0–37, IQR = 5 months, IDR 10–90% = 9 months) and the median time from diagnosis to CUP_MTB_2 was 8 months (range 0–76, IQR = 11 months, IDR 10–90% = 22 months). Time from CUP_MTB_1 to CUP_MTB_2 and from diagnosis to CUP_MTB_2 tended to decrease over the study period, likely reflecting increased frequency of MTB and improved coordination of local and MTB teams over time (data not shown). At time of data analysis, CUP_MTB_2 reports were available for 229/246 (93%) patients, among which 187/246 (76%) had complementary analyses performed, constituting the CUP_diagnostic_population (Fig. 1).

### CUP genomic landscape

**Fig. 2:**
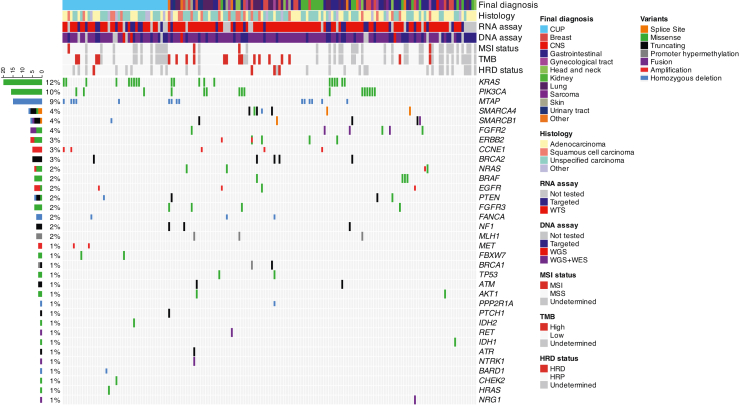
**Molecular landscape of CUPs.** Oncoprint of genomic alterations detected in 165 patients with molecular analyses results. CNS, Central Nervous System; CUP, Cancer of Unknown Primary; TMB, Tumour Mutational Burden; MSI, Microsatellite Instability; MSS, Microsatellite Stable; HRD, Homologous Repair Deficiency; WGS, Whole-Genome Sequencing; WTS, Whole-Transcriptome Sequencing.

Extended genomic profile was available for 165/187 (88%) patients of the CUP_diagnostic_population (Fig. 2 and Supplementary Table S3). Among them, extensive genomic profiling (Whole Genome Sequencing/Whole Exome sequencing) was performed in 108/165 (65%) cases and targeted DNA sequencing in 57/165 (35%) cases. The most frequent molecular alterations identified were variants in *TP53* (74/165, 45%), *CDKN2A/CDKN2B* (33/165, 20% and 20/165, 12%, respectively), *KRAS* (26/165, 16%), *NF2* (18/165, 11%), *TERT* promoter (15/165, 10%), *PIK3CA* (16/165, 10%) and *MTAP* (9%). Targetable molecular alterations were identified in 97/165 (59%) patients, including *ERBB2*, *FGFR2* and *FGFR3* genes variants (Supplementary Figure S3). Among cases with contributive TMB evaluation, 19/125 (15%) tumours had a tumour mutational burden (TMB) > 10 mutations/Mb, 4/124 (3%) exhibited microsatellite instability (MSI), and 4/126 (3%) had Homologous Repair Deficiency (HRD) status validated with a genomic event associated (Supplementary Figure S3). Out of the 51 patients for which mutational signatures could be analysed, 26 were related to precise etiologies, including APOBEC (n = 10/51; 20%), UV exposure (n = 8/51; 16%), and tobacco exposure (n = 8/51; 16%), (data not shown). Among 109 patients who underwent WGS/WES, constitutional cancer predisposition variants were identified in 5/109 cases and involved *TP53* (p169), *BAP1* (p34), *SPINK1* (p176), *BRCA2* (p229) and *CDKN2A* (p75).

### CUP transcriptomic landscape

**Fig. 3:**
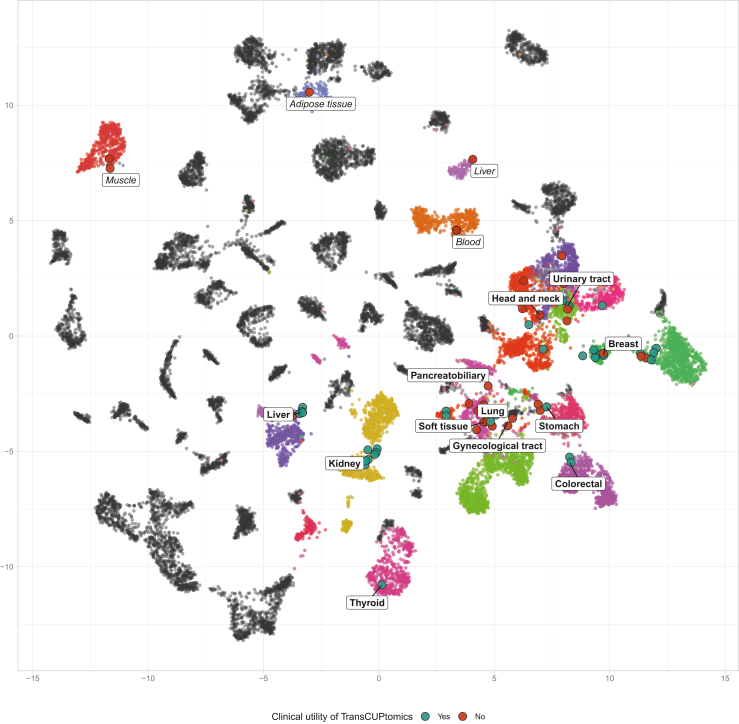
**Transcriptomic landscape of CUPs.** Uniform Manifold Approximation and Projection (UMAP) representing prediction of tissue of origin (TOO) in the cohort of n = 91 samples profiled by total RNA sequencing for which analysis using the TransCUptomics classifier led to high and moderate confidence predictions for TOO. Each CUP sample is represented as a dot, with green dots showing predictions that contributed to the final diagnosis, and red dots showing predictions that had no clinical utility. Predicted tumour types (bold) and normal tissues (italics) are highlighted in colours. Other non-predicted normal tissues and tumour types are coloured in grey. Abbreviations: breast: breast invasive carcinoma; kidney: kidney carcinoma, thyroid: thyroid carcinoma; soft tissue: soft tissue sarcoma; gynecological tract: ovarian and uterine carcinoma; colorectal: colorectal carcinoma; stomach: stomach carcinoma; head and neck: head and neck squamous cell carcinoma; urinary tract: urothelial carcinoma and bladder carcinoma; pancreaticobiliary: pancreatic carcinoma and cholangiocarcinoma; liver: liver carcinoma; lung: lung carcinoma; blood: normal blood; adipose tissue: normal adipose tissue; muscle: normal muscle.

Transcriptomic profiling was performed using total RNAseq for 111 patients and targeted RNAseq for 25 patients. Relevant fusion transcripts were identified in 33/136 (24%) cases, including three specific of sarcoma subtypes (*EWSR1::WT1*, *FUS::DDIT3*, and *PAX3::FOXO1*), three FGFR2 fusions (two *FGFR2::BICC1* and one *FGFR2::PCBD1*), and individual cases with *TPM3::NTRK1* (N = 1), *CCD6::RET* (N = 1), *TFE3::PRCC* (N = 1) and *NRG1::RANK1* (N = 1) fusions (Supplementary Figure S4). Gene expression profiles obtained by total RNAseq were collected to predict the TOO using the TransCUPtomics classifier^17^ in 91/246 (37%) patients (Fig. 3). A putative TOO was predicted with high or moderate confidence in 66/91 (73%) cases. The most frequently predicted TOO were breast carcinoma (n = 13/91, 14%) and lung carcinoma (n = 8/91, 9%) (Fig. 3 and Supplementary Table S4).

### MTB diagnostic orientation

**Fig. 4:**
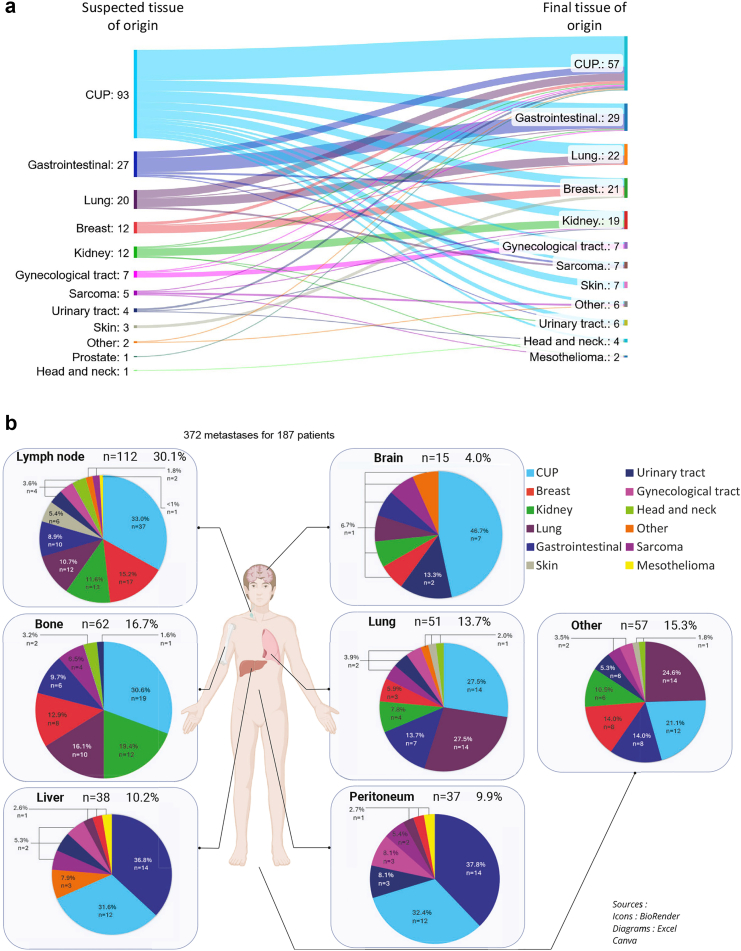
**Diagnostic orientations of CUP_MTB.** a) Suspected tissue of origin (TOO) before CUP_MTB and associated final TOO assigned following CUP_MTB evaluation. b) Final diagnostic orientations (final TOO) assigned following CUP_MTB evaluation according to metastatic sites identified at initial diagnosis. CNS, Central Nervous System; CUP, Cancer of Unknown Primary.

Recommended clinical, pathological and molecular analyses were available and discussed during CUP_MTB_2 for identification of tumour origin in 187 cases (CUP_diagnostic_population). A diagnostic orientation was proposed by CUP_MTB_2 in 130/187 (70%) patients. The TOO identified by the MTB was different from the TOO initially suspected by local teams in 83/130 (64%) cases. The diagnostic orientation was based on the combination of clinical, pathological and molecular characteristics, also in 83/130 (64%) cases (Supplementary Table S1). Among patients for which the TransCUPtomics classifier predicted a specific TOO with high or moderate confidence, this prediction was consistent with the final diagnosis retained by the MTB in 36/66 cases (55%) (Supplementary Table S4 and Supplementary Figure S5).The most frequently identified TOO were gastrointestinal (n = 29/130; 22%), lung (n = 22/130; 17%), breast (n = 21/130; 16%), and kidney (n = 19/130; 15%), and the most frequent tumour subtypes were cholangiocarcinoma (n = 11/130; 8%), colorectal adenocarcinoma (n = 11/130; 8%), and triple-negative breast cancer (n = 11/130; 8%) (Fig. 4a, and Supplementary Figure S6). The most frequent tumor subtypes among patients with a presumed gastrointestinal origin were cholangiocarcinoma (n = 11/29; 38%) and colorectal carcinoma (n = 11/29; 38%), whereas SMARCA4-deficient non-small cell lung cancer was the most common subtype among those with a presumed pulmonary origin (n = 9/22; 41%). For patients with a presumed kidney origin, the most prevalent tumour subtype was undifferentiated renal cell carcinoma (URC) (n = 6/19; 32%). Cervical squamous cell carcinoma and high-grade serous ovarian carcinoma were the most frequent subtypes in patients with a presumed gynecological origin (2/7; 29% respectively) (Supplementary Table S5). In 57/187 (30%) cases, no TOO could be identified despite extensive tumour characterization. Unresolved cases (CUP) did not exhibit specific clinical, histopathological or molecular patterns when compared to resolved tumours, aside from a slight female predominance and their classification as ‘unfavourable’ per ESMO guidelines (Fig. 2 and Supplementary Table S6).

The distribution of identified TOO varied according to metastatic sites at diagnosis (Fig. 4b). For patients with lymph node metastases (n = 112/187), the most frequent TOO were breast (17/112, 15%) and kidney (13/112, 12%). Bone metastases were related mostly to kidney (12/62, 19%) and lung (10/62, 16%) origins, lung metastases to lung (14/51, 27%) and gastrointestinal (7/51, 14%) origins, liver metastases to gastrointestinal origin (14/38, 37%), and peritoneal metastases to gastrointestinal (14/37, 38%) and urinary tract (3/37, 8%) origins. A putative TOO could not be identified in patients presenting with brain metastases in 47% of cases.

### MTB therapeutic orientation

163 out of the 187 patients of the CUP_diagnostic_population were alive at time of CUP_MTB_2 (87%) (CUP_therapeutic_population). The details of recommended therapeutic orientation for each patient within the CUP_therapeutic_population are described in Supplementary Table S7.

A treatment was received in 149/163 (91%) of the cases following CUP_MTB_2 (CUP_treated_population), while 14/163 (9%) were not treated (due to early death) or loss to follow-up.

**Fig. 5:**
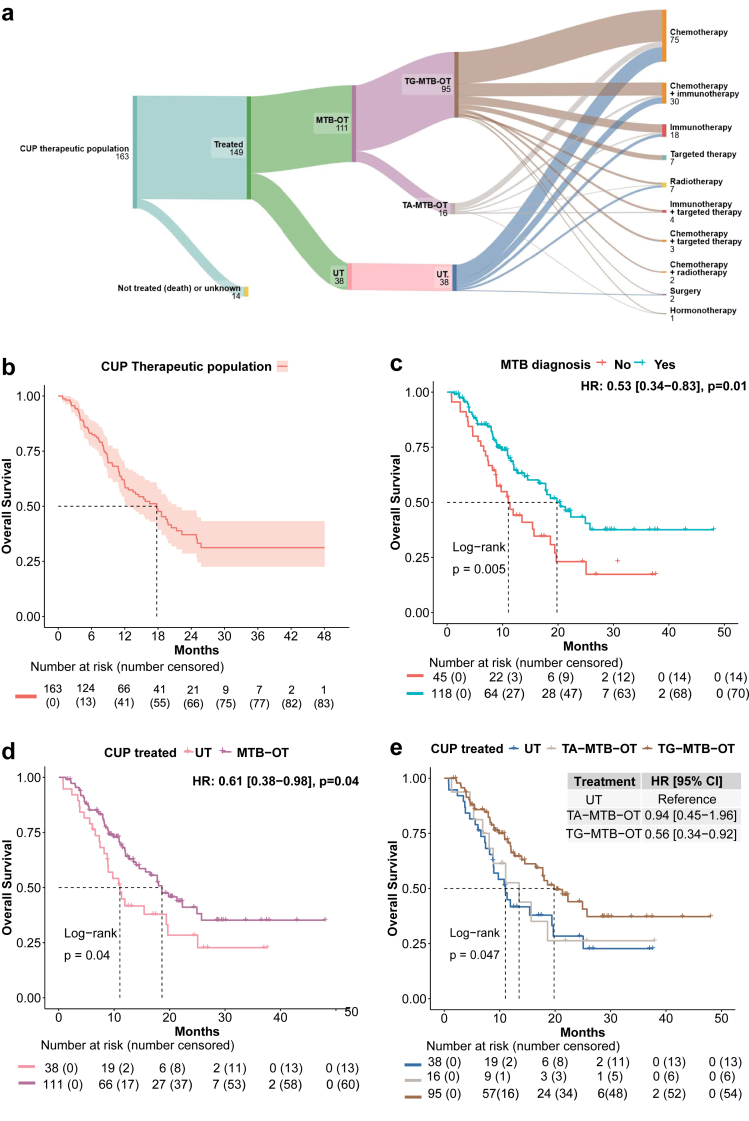
**Therapeutic orientations and outcome following CUP_MTB.** a) Detail of MTB recommendations and treatment received following CUP_MTB. b) Overall survival (OS_2) from CUP_MTB_2 (OS_2) in the CUP_therapeutic_population (N = 163 patients alive at CUP_MTB_2). c) OS_2 in the CUP_therapeutic_population according to the diagnostic impact of CUP_MTB (TOO identified or not identified). d: OS_2 in the CUP_treated_population (N = 149 patients) according to recommendation of MTB-oriented treatment (MTB-OT) or unspecific treatment (UT). e: OS_2 in the CUP_treated_population (N = 149 patients) according to recommendation of tissue-gnostic MTB-oriented treatment (TG-MTB-OT), tissue-agnostic MTB-oriented treatment (TA-MTB-OT) or unspecific treatment (UT).

Among the CUP_treated_population, 111/149 (75%) patients were recommended a treatment oriented towards MTB findings (MTB-OT), including either a tissue-gnostic treatment (TG-MTB-OT) (n = 95/149; 64%) or a tissue-agnostic treatment (TA-MTB-OT) (n = 16/149; 11%). Thirty-eight (25%) patients were recommended non-specific treatment based on international guidelines upon physician's choice (Fig. 5a and Supplementary Table S7). Finally, 132/149 (89%) patients of the CUP_treated_population eventually received a treatment following MTB recommendations, whereas others were treated at the discretion of their referent team.

### Survival analyses

The median overall survival from CUP_MTB_1 (mOS_1) of the CUP_total_population was 10.8 months (Supplementary Figure S7A). The only covariate associated with unfavourable OS_1 in both univariable and multivariable analysis was an ECOG performance status at diagnosis exceeding 1 (Supplementary Tables S8 and Supplementary Figure S7B). The mOS_1 of the CUP_therapeutic population was 17.77 months [12.65–21.32] and the mOS_1 of the CUP_treated population was 17.77 months [12.65–22.31] (Supplementary Figure S8).

The median overall survival from CUP_MTB_2 (mOS_2) for the CUP_therapeutic_population was 17.8 months (Fig. 5b). Among the CUP_therapeutic_population, patients for which the TOO was identified by the MTB had prolonged survival compared to patients for whom the TOO could not be identified (19.8 vs 11 months, HR = 0.53 (95% CI 0.34–0.83), p = 0.01) (Fig. 5c). Among the CUP_treated_population, the mOS_2 of patients treated with MTB-OT was 18.6 months, compared to 11.0 months in patients treated with unspecific systemic treatment (HR = 0.61 [95% CI 0.38–0.98], p = 0.04) (Fig. 5d). The mOS_2 of patients treated with TG-MTB-OT treatment was 19.8 months and the mOS_2 of patients treated with TA-MTB-OT was 13.5 months (HR = 0.56 [95% CI 0.34–0.92]; p = 0.05) (Fig. 5e). Patients who received immune checkpoint inhibitors had prolonged survival as compared to patients who did not (mOS_2 = 24.9 vs 12.0 months, HR = 0.51 [0.31–0.84], p = 0.01) (Supplementary Figure 9).

## Discussion

In this study, we report the diagnostic and therapeutic impact of the French national reference MTB dedicated to patients with CUP during its first three years of existence. To our knowledge, this initiative represents the first multicentre effort aiming at providing nationwide access to clinical expertise, integrated extensive pathological characterization and molecular analyses to guide the management of unselected patients in a real-world setting. CUP_MTB enabled the identification of a likely TOO in 70% of patients who underwent the additional analyses recommended by the MTB. Moreover, 75% of patients eligible to treatment received a specific treatment based on TOO identification or on the detection of a targetable molecular alteration, with a mOS_2 of 18.6 months.

Over the last years, two clinical trials have demonstrated the benefit of molecular profiling to guide the management of patients with CUP.^25^^,^^26^ These trials included highly selected patients (i.e. 1505 patients screened for 438 patients randomized in the CUPISCO trial; 96% of patients with PS < 2 in the Fudan trial), and the benefit of such approaches in the real-world setting remains unknown. Here, we demonstrate that integrated diagnostic characterization including pathological and extensive genomic and transcriptomic profiling is feasible, though challenging, in an unselected CUP population. While 25% of patients died or were lost to follow-up before completion of recommended analyses, 75% benefited from CUP_MTB expert diagnosis. Among them, the putative TOO could be identified in 70% of cases. Of note, the mOS_1 of 10.8 months of our entire cohort is similar to the known survival of patients with CUP in historical series, demonstrating the relevance of our findings.^1^^,^^5^ Reducing turnaround time (TAT) for diagnostic workups and MTB discussion will be essential to prevent early loss of patients and improve the impact of MTB. Operational bottlenecks—such as delays in obtaining patient consent, limited availability of tumour material, or suboptimal coordination can significantly impact TAT between MTB_1 and MTB_2. In addition, sequencing delays may occur in centres with limited technical capacity or low sample throughput. Concrete measures to reduce TAT include better integration of MTB workflows into routine clinical practice, deployment of dedicated coordination teams, improved sample logistics, and, when appropriate, regional consolidation of sequencing in high-throughput reference centres.^29^

With a mOS_2 of 19.8 months for patients receiving a treatment based on the putative TOO, we confirm in a real-world setting the benefit of a tissue-gnostic strategy for CUP management. Benefit of tissue-gnostic approaches in CUP have remained controversial over the last decades.^32^ Earlier randomized studies failed to demonstrate the benefit of tissue-tailored treatment over empiric chemotherapy, whereas the recent Fudan trial showed an improvement of PFS from 6.6 to 9.6 months with tissue–gnostic treatment.^25^ These discrepancies may reflect the heterogeneity of patients and TOO included in these trials, with some cancer subtypes having intrinsic poor prognosis and for which TOO identification may only offer treatments of poor efficacy. In our series, patients treated with tissue–gnostic treatment had prolonged mOS2, despite the over-representation of tumour types classically not related to CUP with favourable prognosis, including biliary tract, lung, and triple negative breast carcinoma. Of note, the strength of our approach compared to previously published methods for TOO identification stands in its integrative aspect combining clinical, pathological and molecular profiles, whereas commonly used approaches only rely on pathology findings or a specific subset of molecular data.^13^^,^^33^ This demonstrates that the classical definition of CUP of favourable and unfavourable prognosis subsets should evolve to consider the availability in a routine setting of expert pathological and molecular assessments.

The CUPISCO trial has recently demonstrated the benefit of treatment guided on targetable molecular alterations after induction chemotherapy in patients with CUP(26). However, in the CUPISCO trial, only 88/326 patients assigned to MGT had a targetable molecular alteration and accessible targeted therapy. In our series, targetable molecular alterations according to OncoKB classification were identified in 59% of patients, and only 8% of these patients were recommended tissue-agnostic treatment. This reflects the lack of access to clinical trials in this population that does not meet classical inclusion criteria for access to drugs not accessible off-label.^34^ The mOS of patients receiving tissue–agnostic treatment was similar to the one of patients treated with unspecific chemotherapy, probably due to the low sample size and the heterogeneity of targets in this population. In contrast, 57 (38%) of patients received ICI either alone or combined to other systemic treatment, and their mOS was significantly prolonged compared to patients that did not receive ICI, confirming the benefit of ICI in CUP as previously reported.^26^^,^^35^

This study could not evaluate the precise diagnostic impact of the different omic techniques used for TOO identification, and particularly of WGS/WES and RNAseq. Indeed, the techniques performed in our study differed depending on available tumour material and accessibility of the techniques over the course of the study. We show that extensive molecular profiling using WGS/WES and RNAseq is helpful in identifying mutational signatures and expression profiles that help TOO identification and that are not provided in large DNA sequencing panels, as recently reported in another study.^36^ These benefits must be counterbalanced by the lack of accessibility of these techniques in various countries and regions. Moreover, they require most of the time frozen tumour material which is not available in all centres. Since November 2023, FFPE tumour samples can be used within the FGMI2025 for CUP profiling. A comparison of the performance and feasibility of the different techniques and types of tumour material will be necessary to determine precisely which molecular techniques will become recommended in the routine setting for CUP management.

Our study also highlights the benefit of reference MTB in the management of CUPs. In our study, the diagnosis proposed by the MTB relied on the combination of clinical, pathological and molecular features in 62% of cases, demonstrating the benefit of a multidisciplinary expertise for CUP diagnostic orientation. The impact of reference networks on the management of rare cancers has been established for several years in other malignancies but never reported for CUP.^37^^,^^38^ In sarcoma for example, reference centres improve the quality of diagnostic and therapeutic procedures, and result in improved overall survival of patients.^39^^,^^40^ For CUP, we demonstrate that expertise in pathology and integration of pathological features with clinical and molecular data enables the identification of a putative TOO in 70% of patients. Of note, the diagnosis of CUP was ruled out without requiring any additional characterization for 31 (11%) patients presented in CUP_MTB, with a major impact on clinical management. This suggests that, like other rare cancers, patients with CUP should be referred to high-volume reference centres or at least be discussed systematically during an expert MTB to benefit from equal access to state-of-the art diagnostic procedures and treatments. Developing local expert CUP_MTB will be necessary to increase the number of patients referred, facilitate sample logistics and shorten delays for final diagnostic and therapeutic orientation.

Our study faces several limitations. First, the number of patients referred to the MTB over three years represents only a fraction of all CUP cases diagnosed over the same period in France based on the expected incidence. Indeed, many centres managing CUP patients in France were not properly informed about this national initiative, and referral of patients to centralized MTB is not part of current international guidelines. Moreover, a number of patients in poor general condition may not have been referred to the MTB by their oncologist as the expected clinical benefit of molecular profiling in such cases is low. Thus, the population presented in this study may be enriched in patients with favourable prognosis. Furthermore, an ongoing medico-economic evaluation within FGMI2025 will provide essential data on cost-effectiveness and guide resource allocation for such organization. For wider implementation, scalability will depend on expanding regional sequencing hubs and increasing expert staffing, both sustained by stable funding. Finally, robust centralized informatics infrastructures, responsible for centralized data storage, processing, and continuous tools improvements, remain critical to ensure reproducibility and facilitate adoption across diverse healthcare systems.

In conclusion, we report here the benefit of CUP_MTB for the clinical management of patients with CUP. We demonstrate that integrative diagnosis and access to MGT is feasible in a real life setting and results in prolonged survival. Development of reference centres and discussion of CUP cases in expert MTB should be recommended as soon as a CUP diagnosis is suspected.

## Contributors

CD, NJ, CLT, SW, and AL designed the study. Data curation and formal analysis were performed by CD, NJ, IG, JF, NF, KN, AL, PG, AH. Investigation and methodology were carried out by NJ, ZN, AH, KN, VC, JS, YA, IS, HB, AP, ADB, JMP, ER, FE, SB, PS, FDF, PB, JW, CT, IG, JF, MK, IB, CLT, SW, NS, PG, HB. Project administration and supervision were managed by CD, SW, KN, IG, and MK. Resources were provided by LC, MM, OM, CLT, SW, PB, IB, MK, VC, PS, SB, AH. Software, validation, and visualisation were handled by AL, PG, NS, NF, CD, AH, IB, CLT, SW, HB, ZN, AL, JMP. The manuscript was drafted by CD, NJ, SW, and AL, and all authors contributed to review and editing. CD and NJ had full access to the raw data. CD, NJ, SW verified the data and SW had final responsibility for the decision to submit the manuscript. All authors approved the final version.

## Data sharing statement

Data collected for this study, including individual participant data and a data dictionary defining each field in the set are available in the supplementary tables.

## Editor note

The Lancet Group takes a neutral position with respect to territorial claims in published maps and institutional affiliations.

## Declaration of interests

SW reports receiving support for attending meetings and/or travel and consulting honoraria from Deciphera and PharmaMar. PS reports receiving honoraria from HTG Molecular Diagnostics, Inivata, ArcherDx, Bristol Myers Squibb, and Roche Molecular Diagnostics; research funding from Roche, AstraZeneca, Novartis, BMS Foundation, Illumina, and Omicure; and support for attending meetings and/or travel from OSE Immunotherapeutics, Illumina, Bristol Myers Squibb, AstraZeneca, and Roche. All other authors declare no competing interests.

## Appendix A Supplementary data

Supplementary Informations

Supplementary Tables

Translated Abstract
