# Supplementary material for: Management and survival of patients with cancer of unknown primary discussed by a French national multidisciplinary tumour board: a retrospective analysis
Source: Lancet Reg Health Eur. 2025 Nov 7;60:101524. doi: 10.1016/j.lanepe.2025.101524 (PMC12639886; doi:10.1016/j.lanepe.2025.101524)
Supplement: Supplementary Informations [file mmc1.pdf]

Supplementary Figures

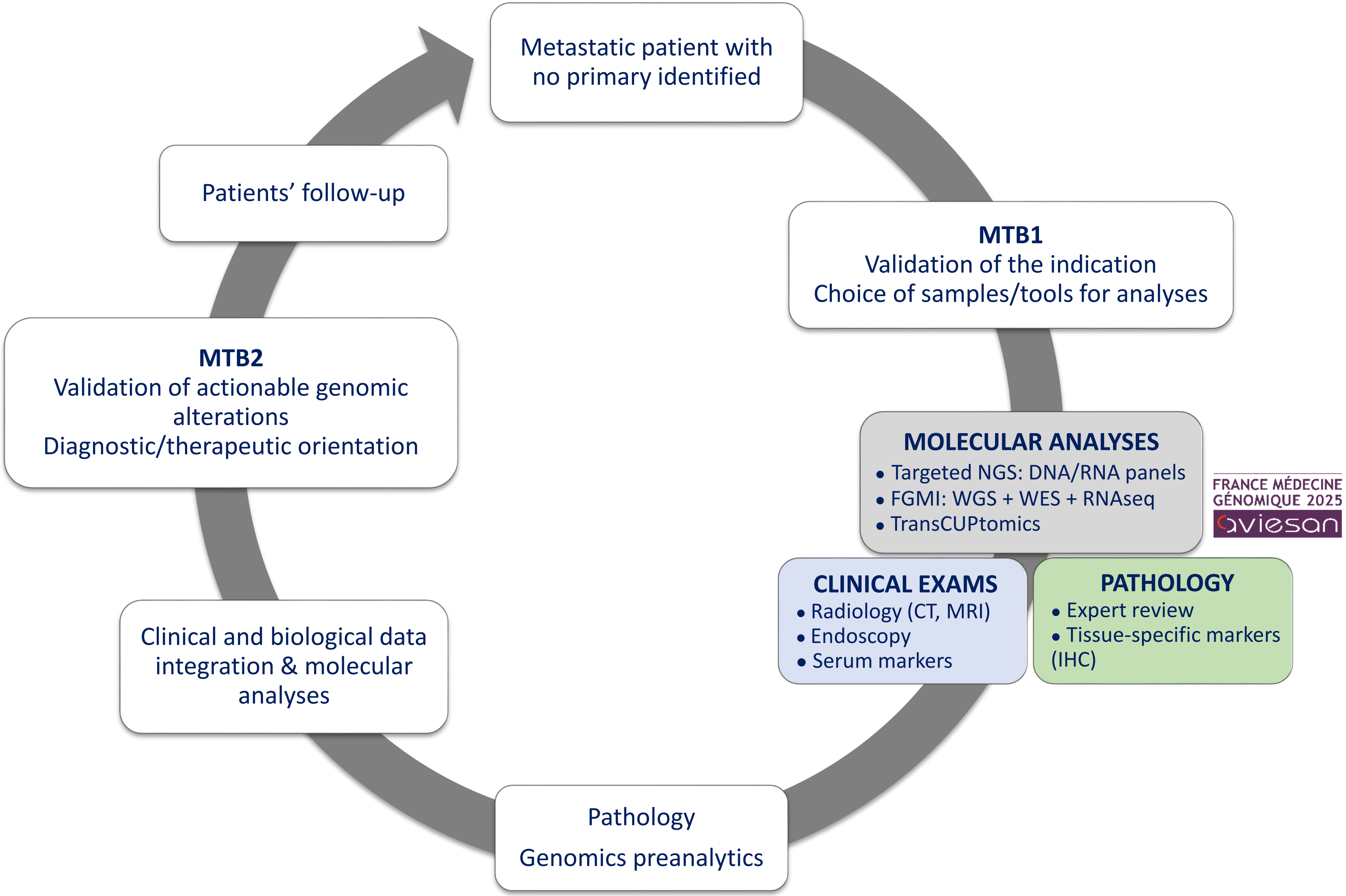

Supplementary Figure 1

a

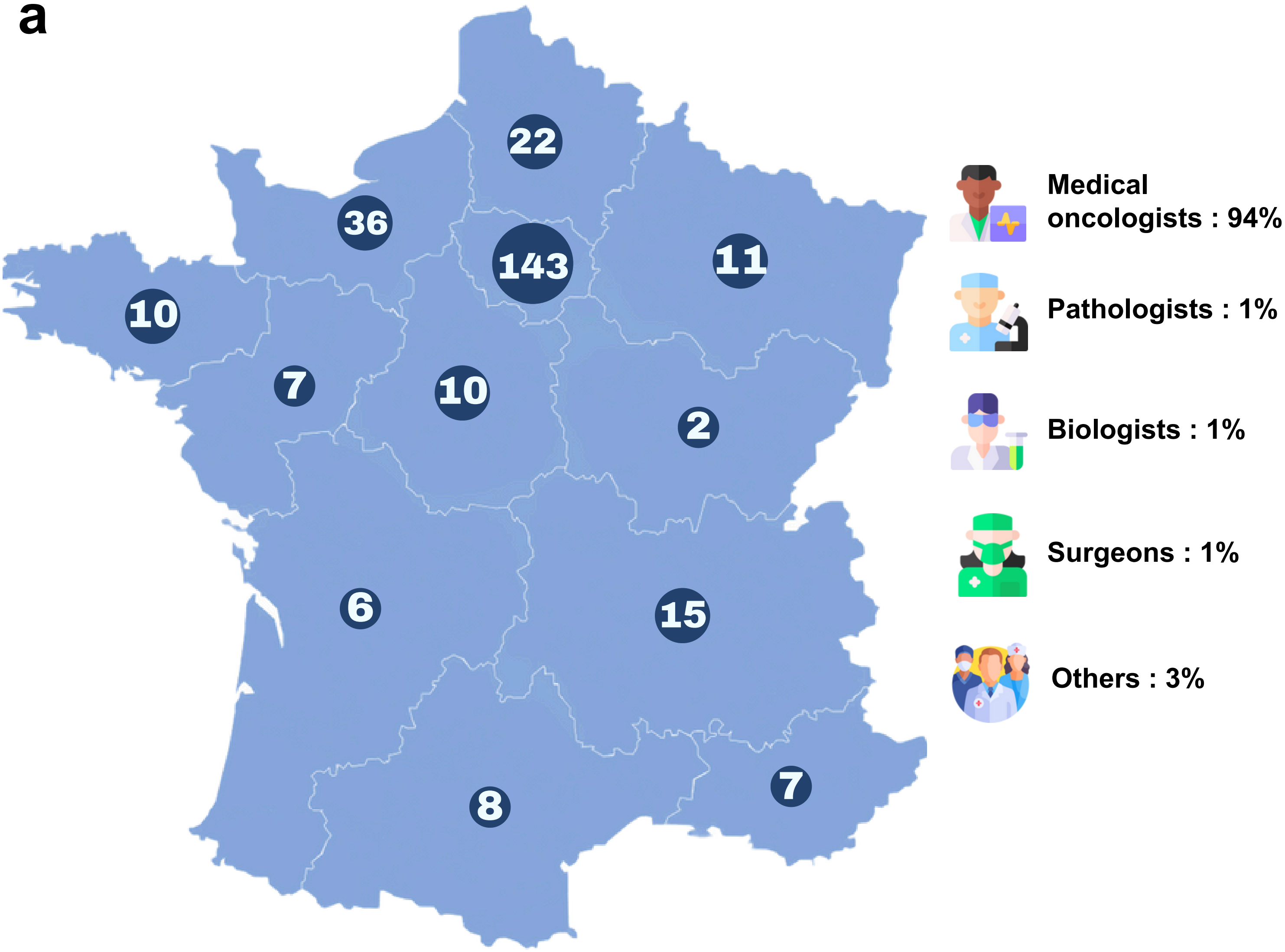

b

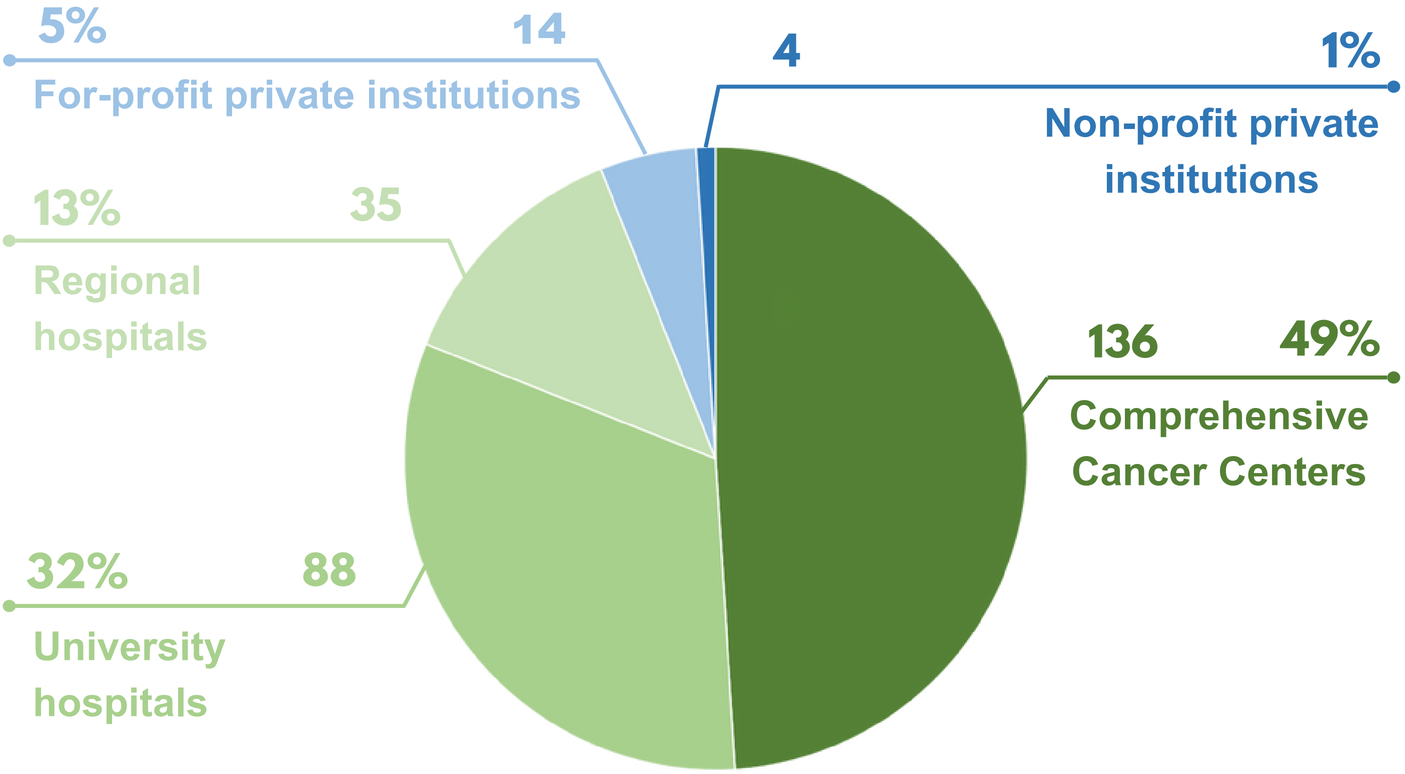

Supplementary Figure 2

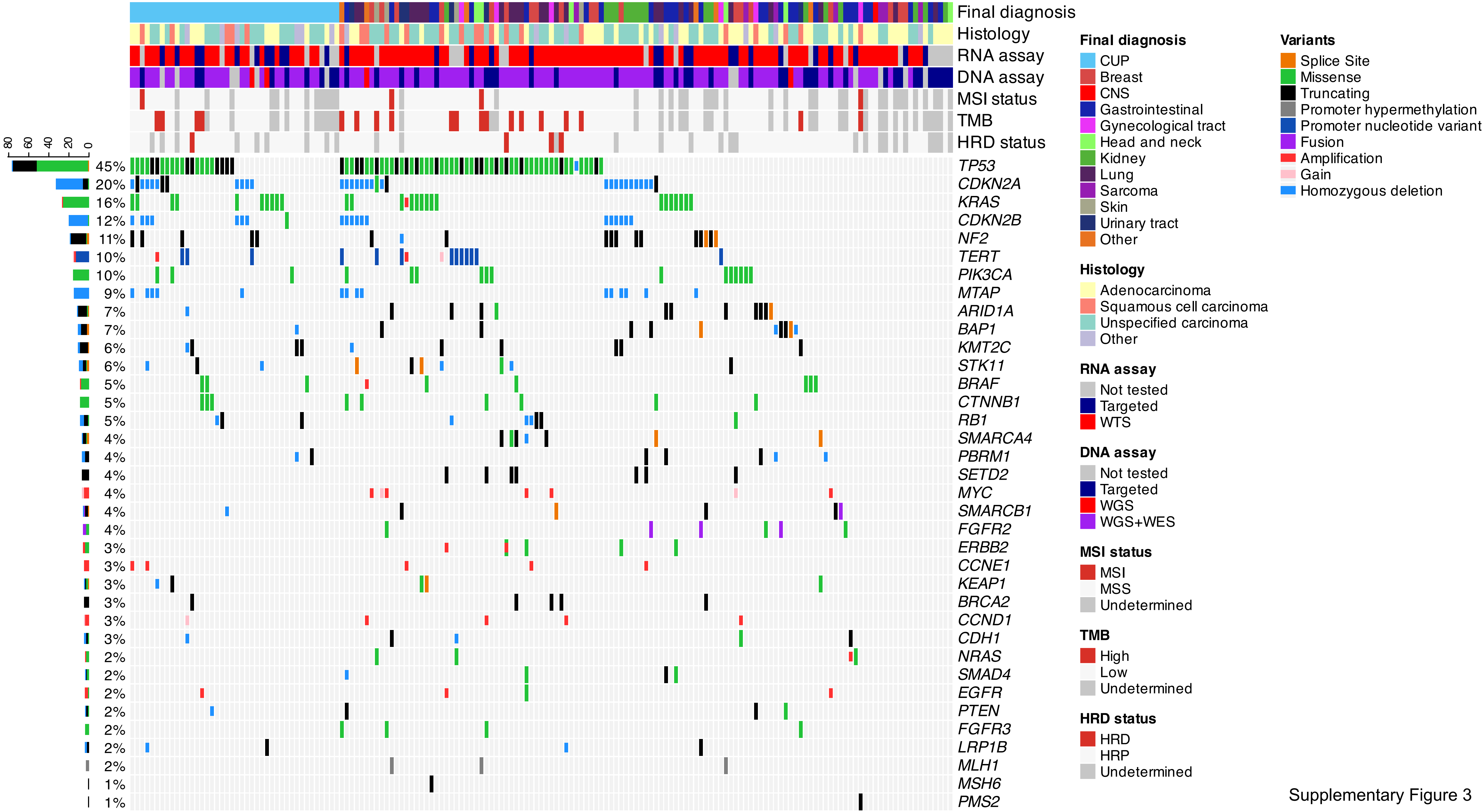

Supplementary Figure 3

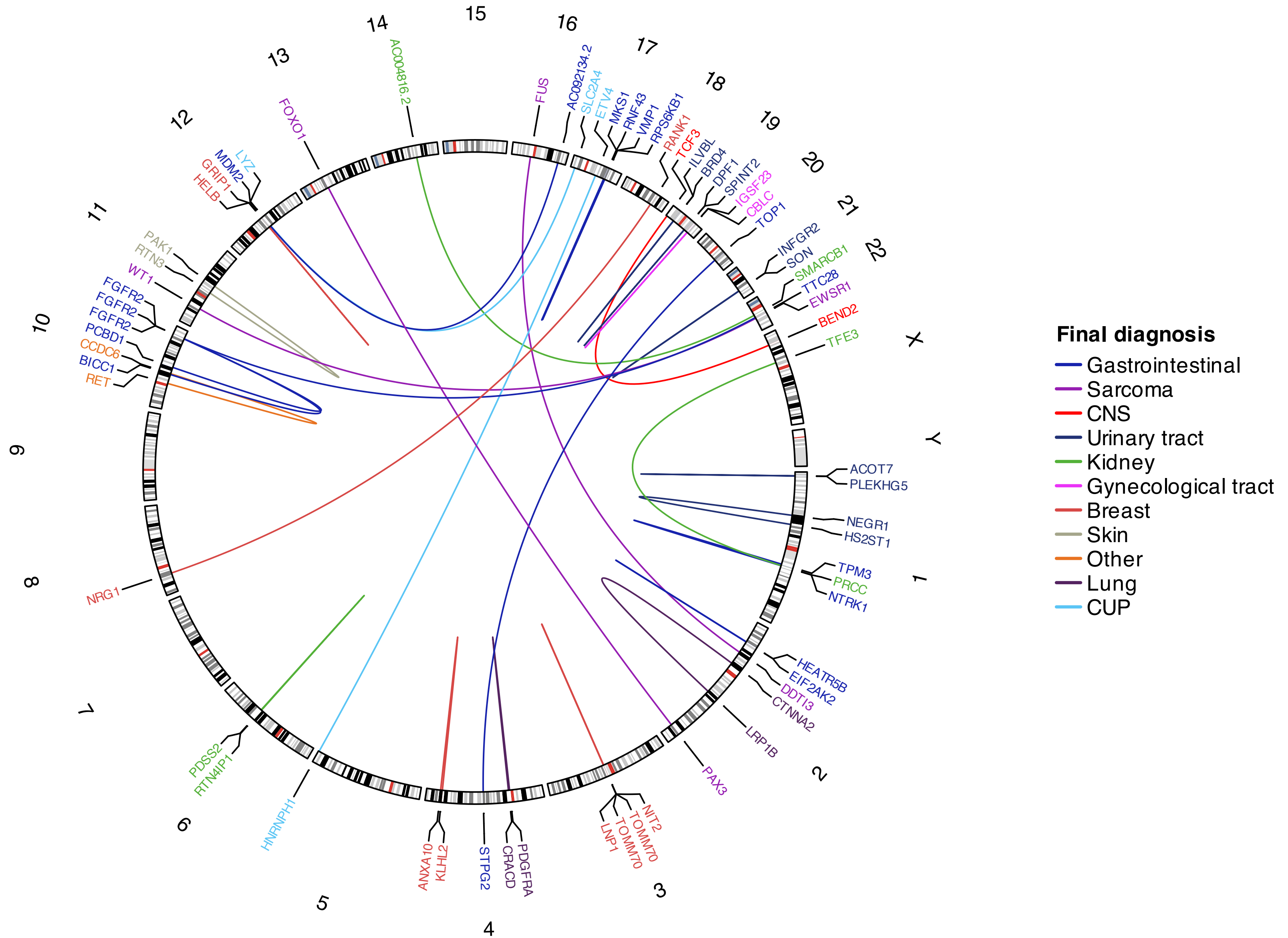

Supplementary Figure 4

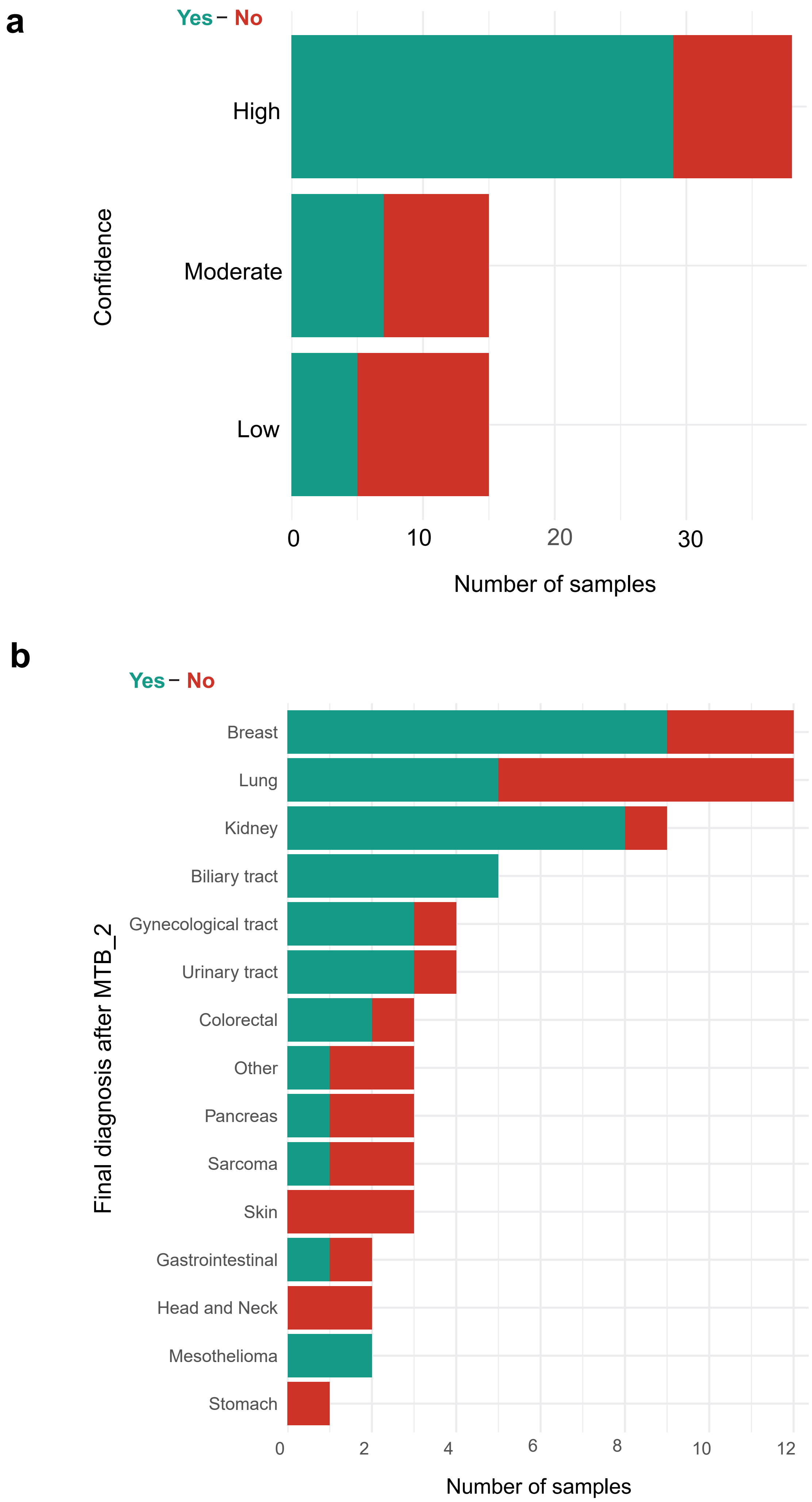

Supplementary Figure 5

Supplementary Figure 6

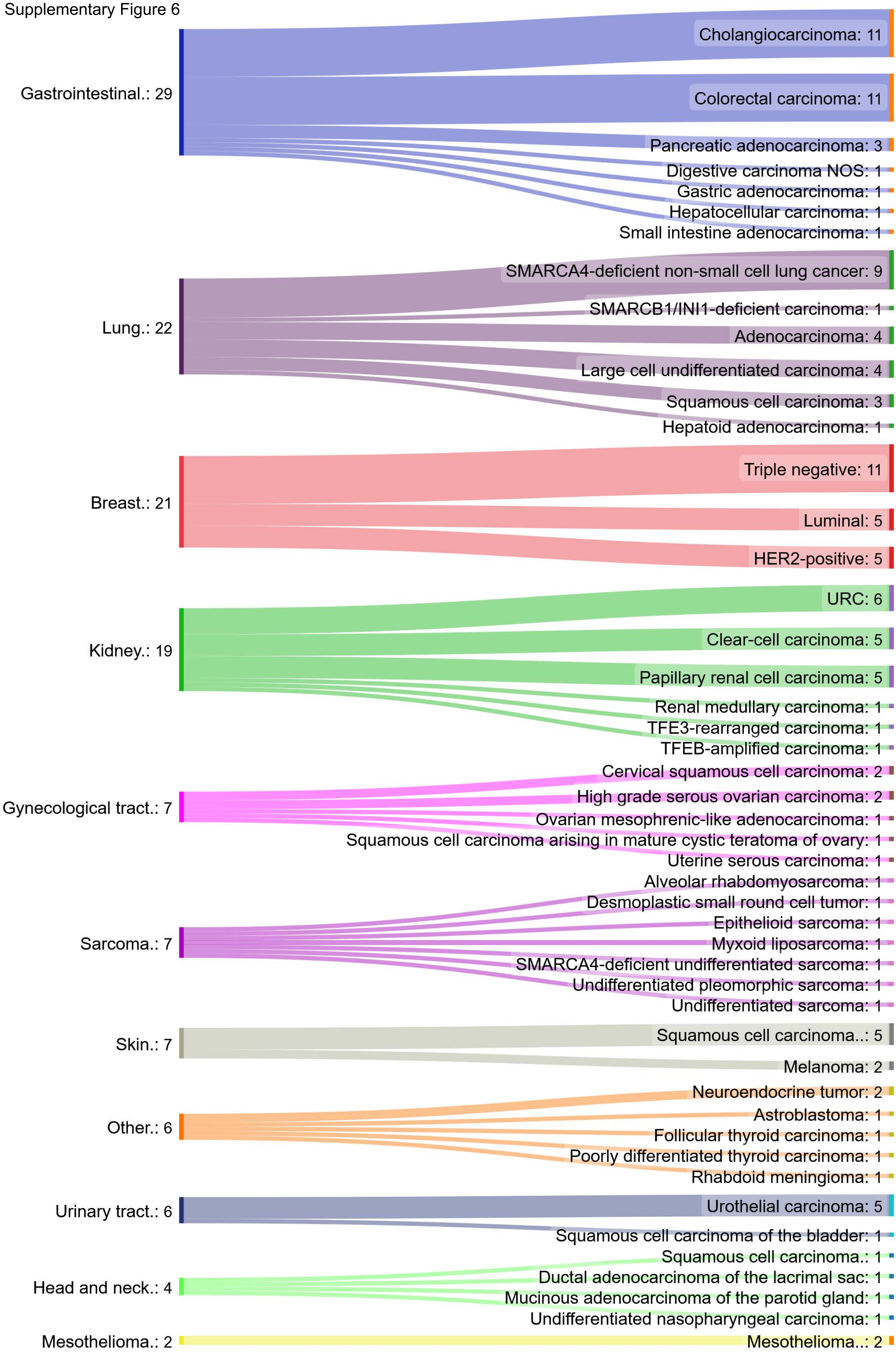

Supplementary Figure 7

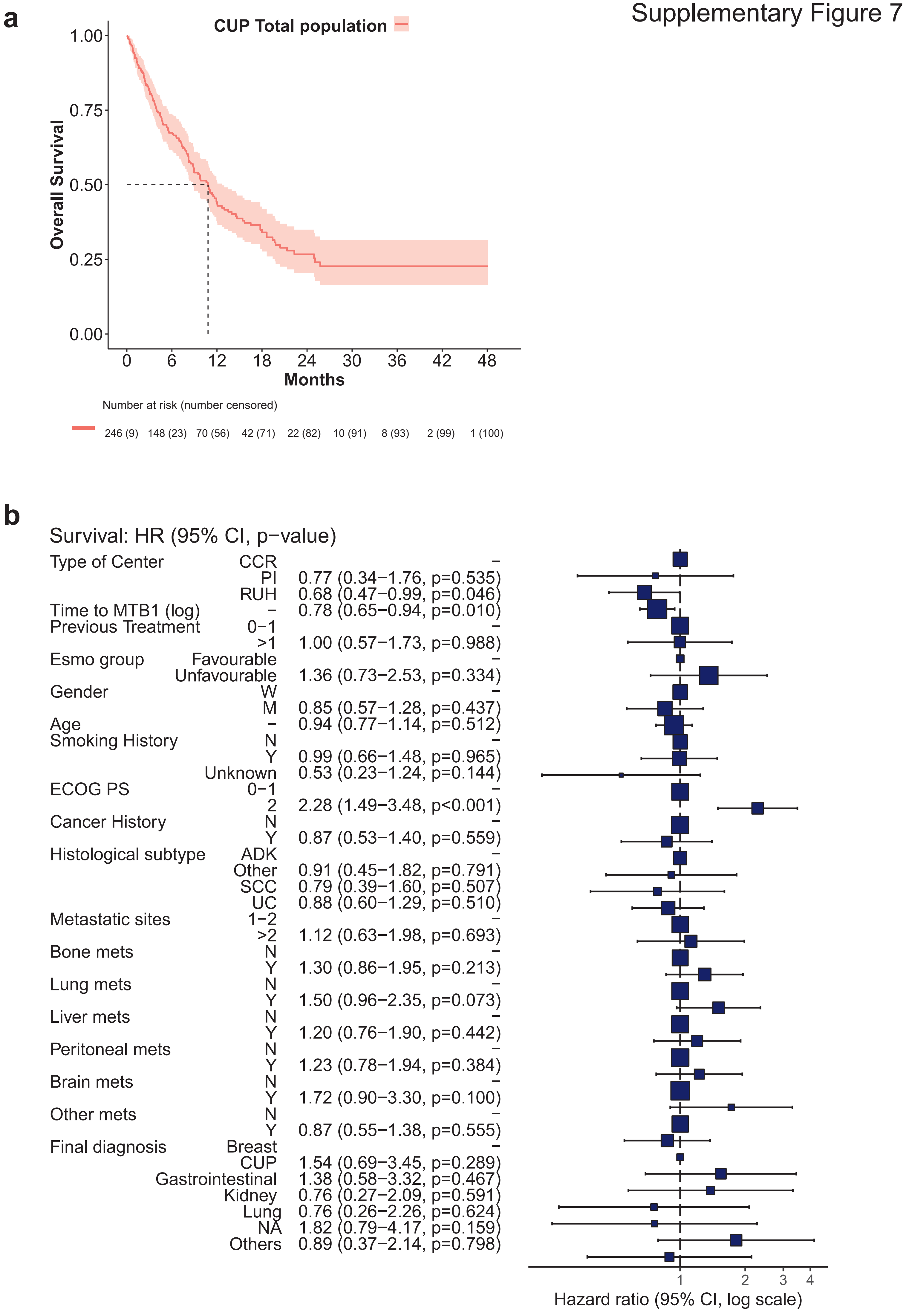

Supplementary Figure 8

a

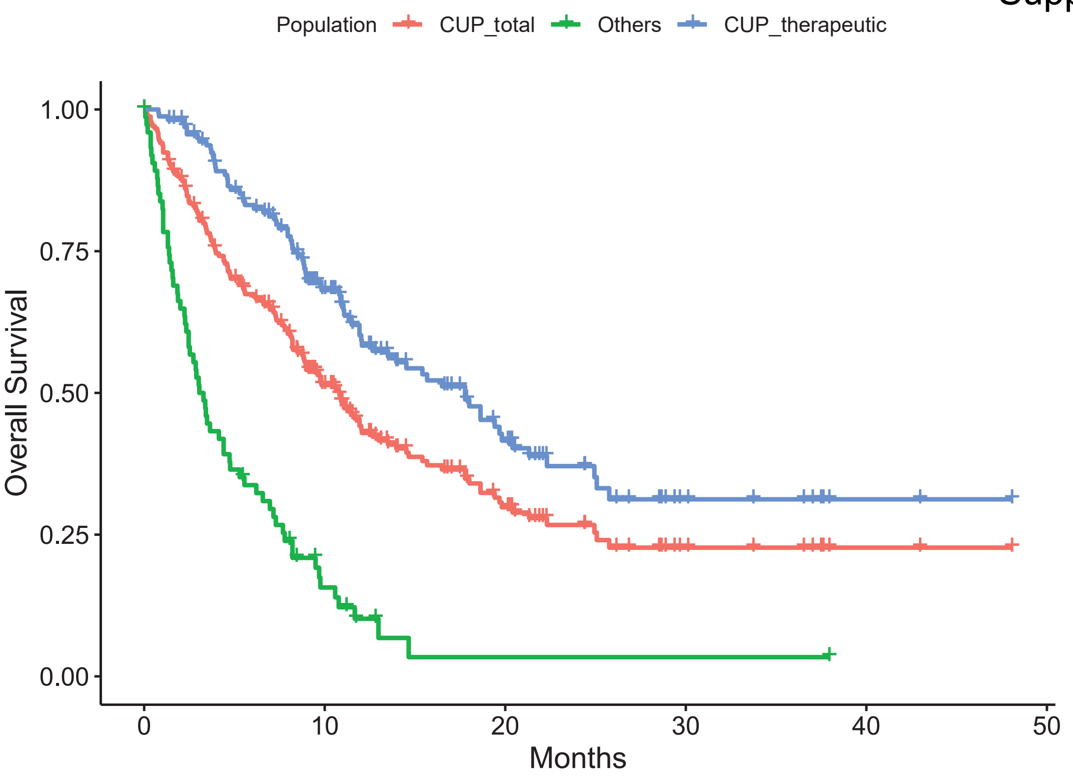

Number at risk

CUP Total	246	95	35	10	2	0
Others	83	9	1	1	0	0
CUP therapeutic	163	86	34	9	2	0

b

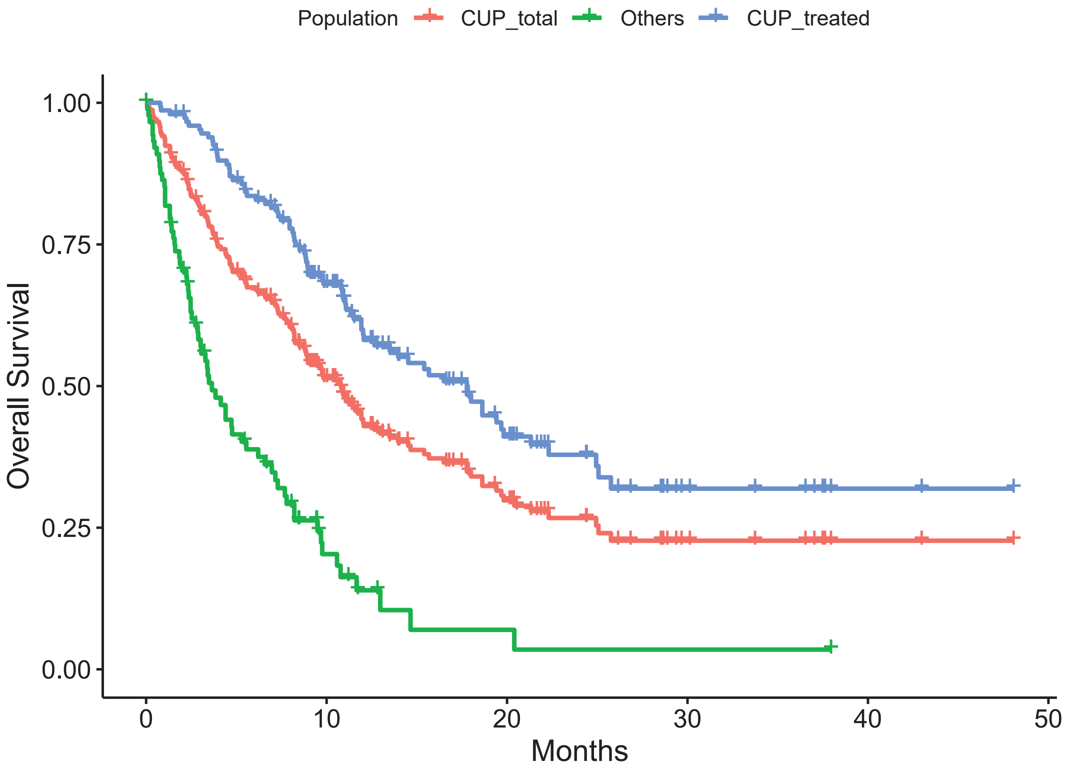

Number at risk

CUP Total	246	95	35	10	2	0
Others	97	10	2	1		
CUP treated	149	85	33	9	2	0

Supplementary Figure 9

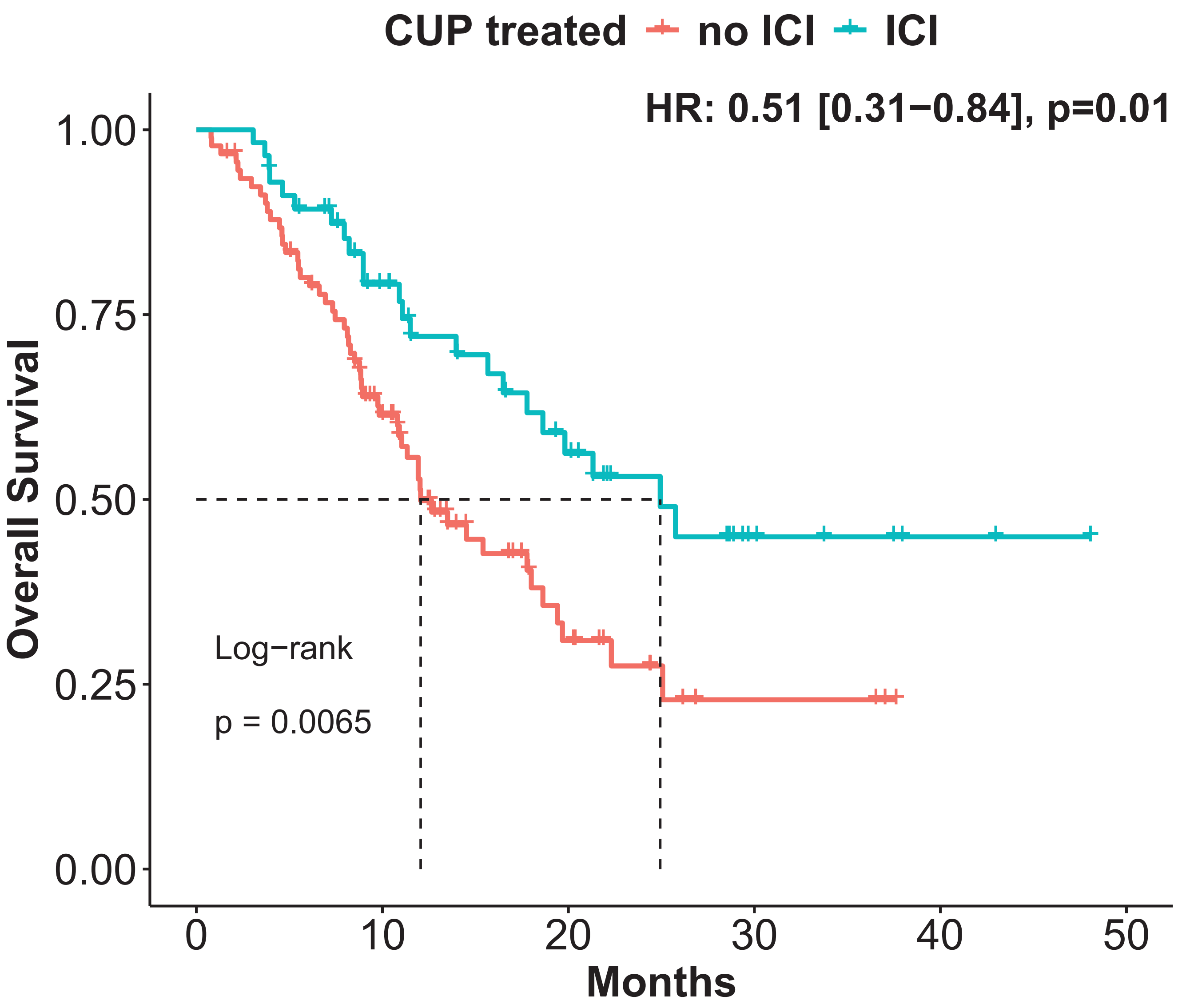

Number at risk (number censored)

no ICI	92 (0)	49 (9)	13 (28)	3 (36)	0 (39)	0 (39)
ICI	57 (0)	36 (10)	20 (17)	6 (28)	2 (32)	0 (34)

Caption for supplementary material

Supplementary Figure 1 – CUP_MTB workflow. Abbreviations: CT: computed tomography; MRI: magnetic resonance imaging; IHC: immunohistochemistry; WES/WGS: Whole Exome Sequencing/ Whole Genome Sequencing; RNAseq: Whole transcriptomic sequencing

Supplementary Figure 2 – CUP_MTB presentation. A) Geographical provenance and addressing specialists of CUP_MTB patients. B) Centers referring patients to CUP_MTB. Sources: Flaticon.com, map adapted from wikimédia.org.

Supplementary Figure 3 – Oncoprint of actionable genomic alterations detected in 163 patients with DNA analyses results, according to OncoKB Therapeutic Level of Evidence 1, 2, 3A, 3B.

Supplementary Figure 4- CIRCOS plot representing pathogenic gene fusions identified by RNAseq.

Supplementary Figure 5: Clinical utility of TransCUPtomics prediction (n=68 samples profiled by RNAseq and for which the MTB had a diagnostic orientation). A: Contribution of TransCUPtomics prediction to the final diagnosis retained by the MTB according to the level of confidence of the prediction. B: Clinical utility of TransCUPtomics according to the final diagnosis retained by the MTB.

Supplementary Figure 6 – Final tissues of origin and detailed diagnoses proposed by CUP_MTB. Abbreviation: URC: undifferentiated renal carcinoma.

Supplementary Figure 7: A: Overall survival from CUP_MTB_1 (OS_1) in the CUP_total_population (n=246 patients). **B:** Impact of clinical and pathological covariates on OS_1 in the CUP_total_population (multivariate analysis). CCC = Clinical Cancer Center; PI = Private Institutions; RUH = Reagional and University Hospital; ADK = Adenocarcinoma; ECOG PS = ECOG Performance Status; SCC = Squamous Cell Carcinoma; UC = Undifferenciated Carcinoma; NA = no diagnosis as patient did not reach CUP MTB_2.

Supplementary Figure 8: A: Overall survival from CUP_MTB_1 (OS_1) in the CUP_total_population (N=246) compared to the CUP_therapeutic population (N=163) and to the population of patients dead or censored before CUP_MTB_2 (others, N=83); B: OS_1 in the CUP_total_population (N=246) compared to the CUP_treated_population (N=149) and to the population of patients not treated or censored after CUP_MTB_2 (others, N=97).

Supplementary Figure 9: Overall survival from CUP_MTB_2 (OS_2) in the CUP_treated_population (N=149 patients) according to immune checkpoint inhibitor (ICI) administration

Supplementary Table S1 – Individual patients' characteristics and management.

Supplementary Table S2 – Complementary analyses recommended and realized within CUP_MTB

Supplementary Table S3 – Genomic alterations identified within CUP_MTB and their actionability according to OncoKB. TRUE = Actionable alteration according to the OncoKB therapeutic Level of Evidence scale (Level 1, 2, 3A and 3B only); TMB = Tumor Mutational Burden; MSI = Microsatellite Instability; MSS = Microsatellite Stable

Supplementary Table S4 – Predictions of tissue of origin based on RNA sequencing and analysis using the TransCUPtomics classifier. For each case, the first prediction using the K-nearest-neighbor (KNN) and Random Forest (RF) classifiers are indicated, as well as the final diagnosis and its level of confidence.

Supplementary Table S5 – Final diagnoses proposed by CUP_MTB in the CUP_diagnostic_population (n=187 patients)

Supplementary Table S6*: Characteristics of patients with unresolved tumors after MTB_2*

Supplementary Table S7 – Treatments recommended and received in the CUP_therapeutic_population (n=163 patients)

Supplementary Table S8: Impact of clinical and pathological covariates on OS_1 in the CUP_total_population (univariate and multivariate analyses). CCC = Clinical Cancer Center; PI = Private Institutions; RUH = Reagional and University Hospital; ADK = Adenocarcinoma; SCC = Squamous Cell Carcinoma; UC = Undifferenciated Carcinoma; NA = no diagnosis as patient did not reach CUP MTB_2.

Supplementary methods

CUP_MTB organization

Between 2020 and 2023, the national Molecular Tumor Board (MTB) for Cancer of Unknown Primary (CUP_MTB) convened bi-weekly via videoconference. The MTB is accessible to all medical specialists in France involved in the management of CUPpatients. For a meeting to be quorate, the mandatory attendees include at least one clinical biologist, one medical oncologist, one pathologist, and one member of the MTB coordination team (MTB_CT). .

Patient cases are referred to the MTB at the request of their medical oncologist. The referral process is initiated by submitting a dedicated Patient Registration Form, containing the patient's comprehensive medical history, to the MTB_CT. The MTB_CT is composed of genomic pathway managers, a role established under the France Genomic Medicine Initiative 2025 (FGMI 2025). The MTB_CT is responsible for documenting and transmitting the MTB's conclusions, follows advancements of analyses, manages the cases discussed during CUP_MTB to ensure all patient referrals are reviewed, and collects all medical variables and follow-up data into the MTB database

CUP_MTB operates as a key component of the FGMI2025 (https://pfmg2025.aviesan.fr/). This integration enables the prescription of advanced genomic analyses, such as Whole Genome Sequencing (WGS), Whole Exome Sequencing (WES), and RNA sequencing (RNAseq), as part of routine clinical care. These analyses are covered by a dedicated national funding mechanism. The FGMI utilizes two national sequencing platforms: SeqOIA for the north-west region of France and AURAGEN for the south-east, French overseas territories, and Corsica [1].

CUP_MTB meetings are structured into two consecutive stages, designated CUP_MTB_1 and CUP_MTB_2.

<u>CUP_MTB_1</u>

During this initial stage, the panel of experts first validates the diagnosis of CUP in accordance with the current European Society for Medical Oncology (ESMO) guidelines [2]. Based on the availability and quality of tumor material, the MTB then recommends any necessary additional clinical, pathological, and/or molecular analyses.

Following CUP_MTB_1, the MTB_CT disseminates a report detailing the discussion and recommendations to the referring physician by email. If molecular analyses within FGMI are requested, the team also undertakes several logistical steps: 1) Ensures the medical prescriptions for genomic testing are formally validated by an authorized prescriber from the relevant platform (SeqOIA or AURAGEN); 2) Provides the referring physician with detailed procedural instructions for collecting blood sample, obtaining patient consent, and shipping tumor and blood samples to the designated facility.

The MTB_CT manages all correspondence between the referring physician, pathologists, biologists, and laboratories to oversee the entire analytical process. The referring physician is also required to inform the MTB_CT of any significant new clinical information that may warrant a re-evaluation in CUP_MTB.

CUP_MTB_2

Once all supplementary pathological, clinical, and molecular results are available, the MTB_CT compiles the complete case file and schedules it for discussion in the CUP_MTB_2 stage. The referring physician is invited to participate in this discussion.

During CUP_MTB_2, the experts review the complete dataset to propose a final diagnostic and therapeutic recommendations. It is important to note that while the MTB provides expert recommendations, the ultimate treatment decision remains the responsibility of the referring clinician.

Upon conclusion of CUP_MTB_2, the MTB_CT sends to the referring physician a final summary of the recommendations, along with the comprehensive FGMI molecular reports, where applicable.

Patients included in the study and follow-up data collection

All adult patients discussed at least once during CUP_MTB between July 2020 and December 2023 were included. Patients' characteristics were obtained from referent physicians at CUP_MTB_1 presentation. Following CUP_MTB_2 conclusions and recommendations, therapeutic strategies were the responsibility of the referent physician. Treatment details and survival status were collected prospectively for at least 2 months after CUP_MTB_2, or until patients' death or loss of follow-up.

Genomic and Transcriptomic analyses

Samples workflow

After validation of the CUP indication and prescription for WGS, WES and RNAseq analyses in the frame of CUP_MTB and FGMI2025, a medical consultation between the referring physician and the patient is organized for the signature of informed consent. Tumor and blood samples are sent to corresponding sites and platforms for extraction of tumor DNA and RNA, as well as constitutional DNA and for sequencing.

Genomic and transcriptomic analyses were performed on the national sequencing FGMI platforms SeqOIA and AURAGEN. Initially, analyses were conducted exclusively on frozen samples. Starting from November 2023, FFPE samples were also included. All tumor samples were required to have at least 30% tumor cell content. Additionally, FFPE samples had to be less than one year old from their collection date.

Whole Exome Sequencing (WES) and Whole Genome Sequencing (WGS) were performed on tumor DNA from cryopreserved tissues and matched germline DNA for detection of single nucleotide variations (SNV) and copy number variations (CNV). Whole transcriptome sequencing (RNA-seq) was

performed on tumor RNA for fusions detection and gene expression profiles were analyzed using the TransCUPtomics classifier[3].

For patients for which the FGMI analyses were not performed, a targeted next generation sequencing DNA or RNA panel was performed locally following local procedures.

French Genomic Medicine Initiative 2025 analyses

Common protocols (library synthesis, sequencing reactions, bioinformatics analyses and genome interpretations) used by AURAGEN and SeqOIA (FMGlabs) were performed as previously detailed[1]. Both FGMI labs performed tumor and germline genome sequencing, and tumor exome and tumor RNAseq using frozen or FFPE cancer tissue samples.

Data processing was performed on local high performance computing facilities using combinations of in-house and on-premise commercial software. French regulation requires certifications for health data management that limits cloud-based software and promotes the control of software and data. Health data management requires complete logging of data access, actions and decisions. Besides, each variant classification is accessible to the whole community of clinical biologists of each FMG lab for future reassessment and internal review, including consensual re-classification.

Interpretation and reporting were also performed as previously described [1]. Variants were reviewed and classified according to the ACMG-AMP guidelines. Variants considered as clinically relevant. The interpretation was not based on an in silico pre-defined target gene panel, but the analysis first focused on genes known to be involved in the pre-indication, and then extended to the whole genome, both coding and non-coding regions.

The results of molecular analyses were discussed during CUP_MTB_2. When required, confirmatory analyses or further biochemical or functional confirmatory diagnostic tests were carried out in the already existing network of clinical laboratories in France.

TransCUPtomics

Transcript per million (tpm) were computed from the raw counts using Gencode v34 to retrieve exon sizes, for n=91 patients with RNAseq analyses with available raw counts data. TransCUPtomics was then used on the tpm values to predict the tissue of origin of each tumor sample [3]. The encoding values from the variational encoder of TransCUPtomics were used to construct the UMAP in two dimensions (R package uwot version 0.1.16).

Oncoprint

Oncoprint was drawn using the ComplexHeatmap package and were performed with the Maftools package for R (version 4.00). Actionable alterations were defined as genomic alterations classified as levels 1-3 by OncoKB [4].

References

1. contributors P. PFMG2025-integrating genomic medicine into the national healthcare system in France. *Lancet Reg Health Eur* 2025; **50**: 101183.
2. Kramer A, Bochtler T, Pauli C, et al. Cancer of unknown primary: ESMO Clinical Practice Guideline for diagnosis, treatment and follow-up. *Ann Oncol* 2023; **34**(3): 228-46.
3. Vibert J, Pierron G, Benoist C, et al. Identification of Tissue of Origin and Guided Therapeutic Applications in Cancers of Unknown Primary Using Deep Learning and RNA Sequencing (TransCUPtomics). *J Mol Diagn* 2021; **23**(10): 1380-92.
4. Chakravarty D, Gao J, Phillips SM, et al. OncoKB: A Precision Oncology Knowledge Base. *JCO Precis Oncol* 2017; **2017**.
