# Supplementary material for: Management and survival of patients with cancer of unknown primary discussed by a French national multidisciplinary tumour board: a retrospective analysis
Source: Lancet Reg Health Eur. 2025 Nov 7;60:101524. doi: 10.1016/j.lanepe.2025.101524 (PMC12639886; doi:10.1016/j.lanepe.2025.101524)
Supplement: Translated Abstract [file mmc3.docx]

**Translated abstract:**

**Editor disclaimer:** This translation in French was submitted by the authors and we reproduce it as supplied. It has not been peer reviewed. Our editorial processes have only been applied to the original abstract in English, which should serve as reference for this manuscript.

**Contexte :** Des études récentes ont montré que les traitements guidés par des analyses moléculaires peuvent améliorer la survie des patients atteints de cancers d’origine inconnue (*CUP : Cancer of Unknown Primary*), mais leur faisabilité et leur bénéfice clinique en pratique réelle restent incertains. En France, une réunion de concertation pluridisciplinaire nationale dédiée aux CUP (RCP nationale CUP) a été mise en place en 2020 pour coordonner les analyses diagnostiques et fournir une expertise centralisée pour l’orientation thérapeutique.

**Méthodes :** Les caractéristiques des patients et des tumeurs, les traitements et les résultats sont recueillis prospectivement. Cette étude rétrospective rapporte l’impact diagnostique et thérapeutique de tous les patients discutés au sein de la RCP nationale CUP entre 2020 et 2023.

**Résultats :** 246 patients atteints de CUP ont été adressés à la RCP nationale CUP (124 femmes et 122 hommes) ; 187 (76 %) ont bénéficié d’une caractérisation pathologique et moléculaire conformément aux recommandations du comité. Le profilage tumoral a permis d’identifier un tissu d’origine probable (*TOO : Tissue Of Origin*) chez 130/187 (70 %) patients. Les TOO les plus fréquents étaient gastro-intestinaux (n=29 ; 22 %), pulmonaire (n=22 ; 17 %), mammaire (n=21 ; 16 %) et rénal (n=19 ; 15 %). 149 (61 %) patients ont reçu un traitement basé sur les recommandations de la RCP nationale CUP. Parmi eux, 111/149 (74,5 %) ont reçu un traitement orienté par la RCP-CUP, incluant un traitement systémique ciblant le TOO probable (n=95, 63,8 %) ou un traitement dirigé contre une altération moléculaire ciblable (n=16, 10,7 %). 38 (25,5 %) patients pour lesquels aucun traitement orienté n’a été recommandé ont été traités par un traitement empirique selon les recommandations internationales. La survie globale médiane (mOS) des patients qui ont eu un traitement orienté était de 18,6 mois, contre 11,0 mois pour les patients ayant reçu un traitement empirique (HR=0,61 ; IC 95 % [0,38-0,98], p=0,04).

**Interprétation :** L’intégration des données cliniques, pathologiques et moléculaires au sein d’une RCP experte est faisable en pratique réelle et améliore la survie d’une grande proportion de patients atteints de CUP. Cette étude souligne l’intérêt des réunions de concertation pluridisciplinaires dédiées et des centres de référence pour améliorer la prise en charge des CUP.

**Financement :** Institut Curie et Plan France Médecine Génomique 2025.
